# Transcriptional profiling reveals glucose-dependent regulation of *COL13A1* mRNA in Pompe patients: Prospect for a novel disease mechanism

**DOI:** 10.1016/j.gendis.2025.101738

**Published:** 2025-06-26

**Authors:** S. Uyttebroeck, D.V. Ngoc, R. Osei, K. Dohr, P. Giron, B.J.H. Dequeker, S. Seneca, K. Sermon, F.J. Hes, A. Gheldof

**Affiliations:** a Vrije Universiteit Brussel (VUB), Universitair Ziekenhuis Brussel (UZ Brussel), Clinical Sciences, Research Group Genetics, Reproduction and Development, Centre for Medical Genetics, Laarbeeklaan 101, Brussels 1090, Belgium; b Department of Paediatrics and Adolescent Medicine, Research Unit of Analytical Mass Spectrometry, Cell Biology and Biochemistry of Inborn Errors of Metabolism, Graz 8010, Austria

Pompe disease, or glycogen storage disease type II (GSD2), is a rare lysosomal storage disorder caused by biallelic pathogenic variants in the acid alpha-glucosidase gene (*GAA*, MIM #606800). The lysosomal enzyme acid alpha-glucosidase (GAA) hydrolyses the 1,4 and 1,6 alpha-glycosidic chemical bonds to break down glycogen into glucose. In Pompe disease, GAA deficiency leads to glycogen accumulation in lysosomes, causing cellular damage. The disease ranges from an infantile-onset form characterized by severe hypotonia, hypertrophic cardiomyopathy, and respiratory failure, often resulting in death within the first year of life, to a milder, late-onset form characterized by progressive muscle weakness, respiratory insufficiency, and significant morbidity.^1^

Enzyme replacement therapy using recombinant GAA has been the mainstay of treatment, substantially improving outcomes, particularly in infantile-onset cases. However, patients who lack residual GAA activity develop neutralizing antibodies against the recombinant enzyme, diminishing its effectiveness. Even in patients with residual GAA activity, the benefits of enzyme replacement therapy can decline over time. These limitations highlight the need for alternative treatment approaches and a deeper understanding of disease mechanisms.^2^

To investigate these mechanisms, we profiled the transcriptional landscape of fibroblasts from four Pompe patients compared with four healthy controls using mRNA sequencing. All material and methods are described in detail in the supplementary data file. Our analysis identified 122 differentially expressed genes (DEGs) (*p* < 0.05), with 74 up-regulated and 48 down-regulated genes in Pompe patients (Fig. 1A). Based on their neuromuscular relevance (as described in Table S1), we selected ten down-regulated genes (*GDNF*, *PIK3R1*, *LYNX1*, *GRIN2A*, *ZNF462*, *COL13A1*, *GABRB3*, *EPHB1*, *KCNMA1*, and *GLRB)* for closer analysis. These genes are presented in a heatmap (Fig. 1B) alongside their expression levels in five tissues of interest. The heatmap for the top 50 DEGs can be found in Fig. S1. Gene set enrichment analysis indicated involvement in pathways critical to muscle structure and function, such as extracellular matrix organization and collagen metabolism (Fig. 1C). To validate these findings, we expanded the cohort, confirming consistent differential expression of *PIK3R1*, *GABRB3*, *COL13A1*, and *KCNMA1* in additional patients' fibroblasts (10 controls and 16 patients) (Fig. S2).

**Figure 1:**
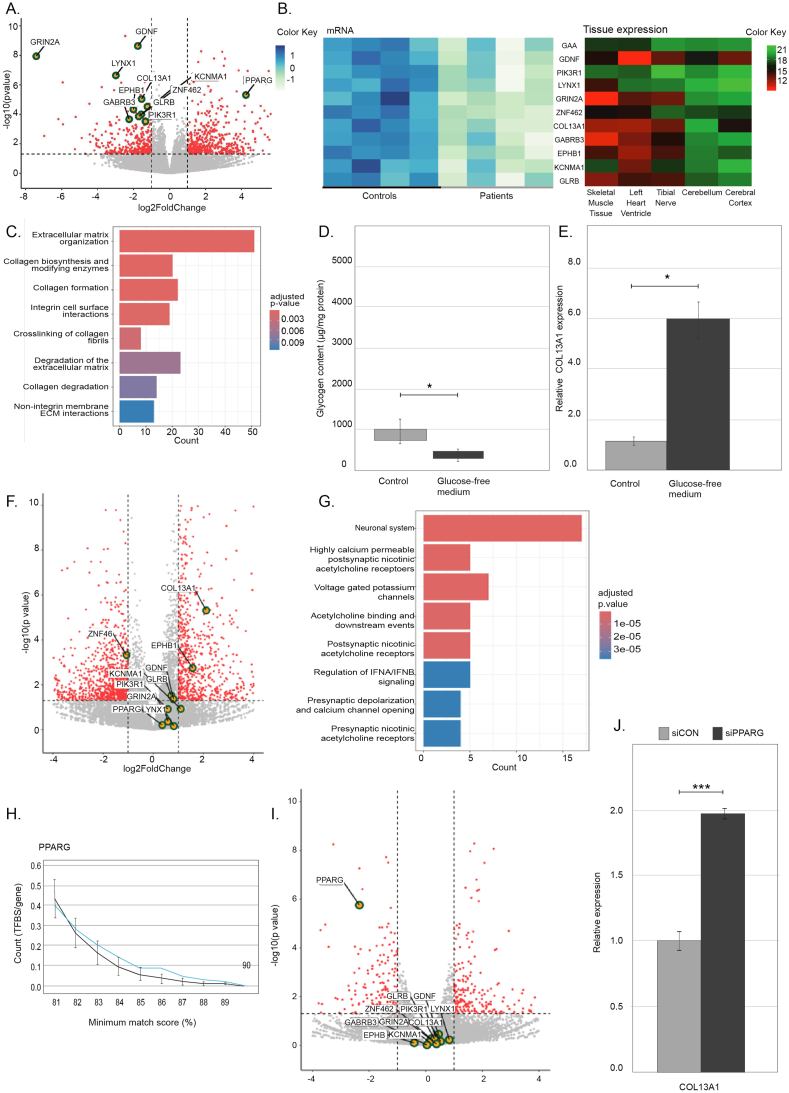
Transcriptional profiling in Pompe disease. **(A)** Differential expression analysis of mRNA sequencing in Pompe patients' fibroblasts compared with controls, with red point representing differentially expressed genes (DEGs) (*p* < 0.05), either down-regulated (log_2_ fold change < −1, upper left) or up-regulated (log_2_ fold change >1, upper right) (*n* = 4 per group). **(B)** Heat map of selected DEGs between Pompe patient and control fibroblasts, with a heatmap of expression levels in five tissues as per the GTEx Portal. **(C)** Gene set enrichment analysis of the 122 DEGs using ReactomePA, showing a prominent role in the organization of the extracellular matrix and collagen metabolism. **(D)** Glycogen content normalized by protein amount in patient samples (*n* = 4) cultured in complete medium (gray) versus glucose-free conditions (black). **(E)***COL13A1* expression in Pompe patients' fibroblasts (*n* = 4) after 96 h glucose depletion. **(F)** Differential expression analysis of mRNA sequencing after glucose depletion experiments in Pompe patients' fibroblasts (*n* = 4). **(G)** Gene set enrichment analysis of the DEGs under glucose-depleted conditions, suggesting a prominent role in the regulation of neuromuscular functionality. **(H)** PPARγ binding site enrichment analysis, comparing the number of PPARγ binding sites in the promoters of DEGs in the Pompe mRNA sequencing dataset (blue line) to 1000 random gene sets (black line). **(I)** Differential expression analysis of mRNA sequencing of cells transfected with siRNA targeting PPARγ versus a control siRNA (*n* = 4). **(J)***COL13A1* expression in patient 5 after transfection with a control siRNA and siRNA targeting *PPARG*.

Among these DEGs, the collagen type XIII alpha-1 gene (*COL13A1*, MIM #120350) was significantly down-regulated. *COL13A1* encodes collagen type XIII (COL13A1), which stabilises the clustering of acetylcholine receptors (AChRs) at the neuromuscular junction in muscle cells. While typical collagens contribute to the extracellular matrix, COL13A1 integrates into muscle cell membranes and physically interacts with AChRs. We hypothesize that *COL13A1* down-regulation in Pompe patients may disrupt neuromuscular junction function.

Bi-allelic pathogenic variants in *COL13A1* are linked to congenital myasthenic syndrome type 19, a disorder with overlapping symptoms, including muscle weakness and hypotonia. The phenotypic similarity hints at a partial role of COL13A1 in the etiology of Pompe disease.^3^

Our analysis showed that *COL13A1* expression was modulated by glucose metabolism. Under glucose-free culture conditions, Pompe fibroblasts exhibited decreased glycogen content (Fig. 1D) and increased *COL13A1* mRNA levels on real-time quantitative PCR (Fig. 1E and Table S2) and confirmed by mRNA sequencing (*n* = 4) (Fig. 1F). This indicates that *COL13A1* deregulation in Pompe patients is glucose-dependent, emphasizing the importance of metabolic regulation in the disease. Pathway enrichment analysis under glucose-depleted conditions highlighted disruptions in neuronal processes, including presynaptic AChR signaling (Fig. 1G).

These findings align with known metabolic disturbances in Pompe disease, where impaired glycogen breakdown in lysosomes affects cytoplasmic glycogen levels and alters glucose-responsive gene expression. This study suggests that targeting glucose metabolism may be a potential therapeutic strategy, as current enzyme replacement therapy does not address cytoplasmic glycogen pools. Therapeutic approaches that reduce cytoplasmic glycogen, such as dietary glucose restriction or glycogen synthesis inhibitors, could help normalize *COL13A1* expression and improve muscle function.

To explore the transcriptional regulation of *COL13A1* and other DEGs, we conducted *in silico* promoter analysis, focusing on potential transcription factor binding sites. Binding site enrichment analysis is shown in Figure 1H for peroxisome proliferator-activated receptor gamma (PPARγ) and other transcription factors in Figure S3. The number of binding sites for each transcription factor in the promoters of DEGs in the Pompe mRNA sequencing dataset was compared with 1000 random gene sets of equal size. We identified a significant enrichment of binding sites for PPARγ, a transcription factor involved in lipid metabolism, glucose homeostasis, and adipogenesis, suggesting a possible regulatory role. Importantly, *PPARG* expression was significantly up-regulated in Pompe patient fibroblasts compared with controls, further supporting its involvement in disease-associated transcriptional changes. Despite this, siRNA-mediated knockdown of *PPARG* in Pompe fibroblasts did not consistently alter *COL13A1* expression across all four patient samples, as shown on the volcano plot (Fig. 1I). However, in one patient, we observed a notable increase in *COL13A1* expression following PPARγ knockdown (Fig. 1J), indicating that while PPARγ may not universally regulate *COL13A1*, it could have a differential role depending on individual patient contexts. This variability underscores the potential for personalized therapeutic strategies targeting PPARγ pathways in subgroups of Pompe patients.^4^ All patient and control demographics are provided in Table S3.

The possible overlap between Pompe disease and myasthenic syndrome type 19 due to altered *COL13A1* expression may lead to new therapeutic options. Certain types of myasthenic syndromes are treated with salbutamol, a β2-adrenergic receptor agonist commonly used to relieve bronchospasm in conditions like asthma and chronic obstructive pulmonary disease. Salbutamol functions primarily by stimulating β2-adrenergic receptors through increased cyclic AMP levels, which result in smooth muscle relaxation and bronchodilation. In the context of myasthenic syndromes, salbutamol has been observed to induce significant clinical improvements, which may be attributed to its effect on enhancing the stabilization of AChR clustering at the neuromuscular junction. This study rationalizes the use of β2-adrenergic receptor agonists as a therapeutic avenue and warrants the need for further research in this field.^5^

In conclusion, our study identifies *COL13A1* down-regulation as a novel mechanism that may contribute to the neuromuscular dysfunction observed in Pompe disease. By linking glycogen metabolism with gene expression changes at the neuromuscular junction, our findings provide new insights into the complex pathophysiology of this disorder, offering therapeutic opportunities. Further research is warranted to validate these findings in muscle tissues and to explore how these mechanisms can be exploited to develop more effective, personalized treatment strategies for Pompe patients.

## Ethics declaration

This study received approval from the Institutional Ethical Commission of our hospital UZ Brussel, Belgium. (Approval No. EC-2018-379). Written informed consent for sample collection and subsequent analysis was obtained from all participants.

## CRediT authorship contribution statement

**S. Uyttebroeck:** Visualization, Investigation, Formal analysis, Writing – original draft. **D.V. Ngoc:** Investigation, Writing – original draft, Formal analysis, Visualization. **R. Osei:** Software, Investigation. **K. Dohr:** Investigation. **P. Giron:** Writing – review & editing, Resources, Funding acquisition. **B.J.H. Dequeker:** Supervision. **S. Seneca:** Supervision. **K. Sermon:** Resources. **F.J. Hes:** Supervision, Resources, Funding acquisition, Writing – review & editing. **A. Gheldof:** Project administration, Funding acquisition, Writing – review & editing, Supervision, Conceptualization, Formal analysis, Software, Methodology.

## Funding

This research was supported by funding from the Wetenschappelijk Fonds Willy Gepts of the UZ Brussel, Belgium.

## Conflict of interests

The authors declare that they have no known competing financial interests or personal relationships that could have appeared to influence the work reported in this paper.
