# Supplementary material for: Transcriptional profiling reveals glucose-dependent regulation of COL13A1 mRNA in Pompe patients: Prospect for a novel disease mechanism
Source: Genes Dis. 2025 Jun 26;13(1):101738. doi: 10.1016/j.gendis.2025.101738 (PMC12495276; doi:10.1016/j.gendis.2025.101738)
Supplement: Multimedia component 1 [file mmc1.docx]

## Supplementary: material and methods

Ethical Approval: This study received approval from the Institutional Ethical Commission of our hospital UZ Brussel, Belgium. (EC-2018-379).

Dermal fibroblasts cell lines: Human dermal fibroblasts were obtained from patients suspected of having Pompe disease, that were submitted to UZ Brussel, Centre for Medical Genetics, for enzymatic diagnostic testing. Control fibroblasts from healthy donors were cultured from skin punch biopsies as described by Aasen et al. [1]. Dermal biopsies were collected in phosphate buffered saline (PBS) and subsequently cut in pieces of approximately 10mm². These fragments were placed in a culture flask with Ham’s F12 Nutrient Mix (Gibco), supplemented with 20% fetal bovine serum, 1% L-Glutamine and 1% penicillin-streptomycin (Gibco) in a humidified incubator at 37°C and 5% CO2. The skin fragments were removed after sufficient fibroblast outgrowth. Further passaging was done by washing the cells with PBS prior to trypsinization.

RNA extraction: Total RNA was extracted from fibroblasts or C2C12 cell pellets (stored at -80°C following collection) using the PureLink RNA Mini Kit (ThermoFisher Invitrogen) with the Trizol Plus Total Transcriptome Isolation protocol provided by the manufacturer, and eluted in RNAse-free water. All samples were stored at -80°C until further use. RNA concentrations were quantified using a spectrophotometer (NanoDrop-2000; Thermo Fisher Scientific).

Real time quantitative PCR (RT-qPCR): Complementary DNA (cDNA) was synthesized from RNA samples using the iScript cDNA Synthesis Kit (Bio-Rad). RT-qPCR was performed using the LightCycler 480 SYBR Green I Master Kit (Roche). Specific primers for our genes of interest and housekeeping genes are included in Supplementary Table 2.

RNA sequencing: Transcriptome analysis was performed using the TruSeq RNA v1 kit (Illumina) with total RNA extracted from fibroblasts of healthy controls and Pompe patients as input material. Sequencing was done on an Illumina Hiseq 1500 or NovaSeq 6000 machine. Read alignment was performed by using STAR aligner [2]. The total read number of each sample was aimed at 50 million, with an average amount of reads of 46 million across all samples. The average alignment percentage to the transcriptome across all samples was 89.5% with a minimum of 87.7 and a maximum of 91.3%. Read length was 100bp with a paired end sequencing modality. Differential expression analysis was performed using the R/DESeq2 package [3]. Tissue expression data for each gene was obtained from the GTEx Portal, and tissue expression for the miRNA from TissueAtlas[4]. Gene ontology analysis on mRNA sequencing datasets was performed with Database for Annotation, Visualization and Integrated Discovery (DAVID; [5]) functional annotation analysis, using the default parameters. Further pathway analysis comparisons was performed using the R/Bioconductor GAGE [6] and Pathview package [7]. Gene set enrichment was performed using the enrichPathway function of the R package ReactomePA.

GAA enzymatic assay: Biochemical measurement of GAA enzymatic activity was done using a fluorescence-based assay 4-methylumbelliferyl-α-D-glucopyranosid (4-MU) at pH 4. Fluorescence was measured on the PerkinElmer VICTOR Nivo Multimode Microplate Reader (excitation 355/40 nm, emission filter 460/30 nm, duration: 20 ms).

Glycogen depletion: Fibroblasts from healthy controls and Pompe patients were grown in glucose-free Dulbecco's modified Eagle’s medium with 20% foetal bovine serum for 4 days after confluence prior to collection. The depletion was verified by measuring the glycogen content in the samples using a coupled enzyme glycogen assay kit (MAK016, Sigma-Aldrich), and measured on the PerkinElmer VICTOR Nivo Multimode Microplate Reader (530/30 nm, 12000 ms).

Promoter analysis: Promotor analysis was performed with an in-house developed R-script based on the R/JASPAR2022 package to reference the DNA binding [8]. We used this to identify potential PPARgamma binding sites in the promoter region (1500 basepairs upstream and downstream of the transcription start site) of the differentially expressed genes from our mRNA sequencing dataset, given a minimum match score of 80%. The number of Transcription Factor Binding Sites (TFBS) found in this given dataset was compared to 1000 randomly generated gene sets with an equal number of genes.

siRNA-mediated knockdown of PPARG: Patients' fibroblasts were plated at approximately 320,000 cells per well in complete medium in 6-well plates. These cells were transfected using Lipofectamine RNAiMAX (Invitrogen) with either siGENOME SMARTPool human PPARG siRNA (Dharmacon) or siGENOME non-targeting Control Pool #1 siRNA (Dharmacon) as control, both reconstituted in 5X siRNA buffer (Dharmacon). The final siRNA concentration was 20 nM, with 7.5 μL Lipofectamine per well. Cells were refreshed with medium after 16 hours and lysed 72 hours post transfection.

Statistical analysis of RT-qPCR data: Pairwise comparison of samples was performed using Wilcoxon test, with statistical significance defined at p < 0.05 unless otherwise specified. When appropriate, multiple testing correction was performed using the Bonferroni method.

[1] T. Aasen and J. C. I. Belmonte, “Isolation and cultivation of human keratinocytes from skin or plucked hair for the generation of induced pluripotent stem cells,” Nat. Protoc., vol. 5, no. 2, Art. no. 2, Feb. 2010, doi: 10.1038/nprot.2009.241.

[2] A. Dobin et al., “STAR: ultrafast universal RNA-seq aligner,” Bioinforma. Oxf. Engl., vol. 29, no. 1, pp. 15–21, Jan. 2013, doi: 10.1093/bioinformatics/bts635.

[3]M. I. Love, W. Huber, and S. Anders, “Moderated estimation of fold change and dispersion for RNA-seq data with DESeq2,” Genome Biol., vol. 15, no. 12, p. 550, 2014, doi: 10.1186/s13059-014-0550-8.

[4]“Distribution of miRNA expression across human tissues | Nucleic Acids Research | Oxford Academic.” Accessed: Jan. 24, 2024. [Online]. Available: <https://academic.oup.com/nar/article/44/8/3865/2467026>

[5]“Systematic and integrative analysis of large gene lists using DAVID bioinformatics resources | Nature Protocols.” Accessed: Jan. 24, 2024. [Online]. Available: <https://www.nature.com/articles/nprot.2008.211>

[6] W. Luo, M. S. Friedman, K. Shedden, K. D. Hankenson, and P. J. Woolf, “GAGE: generally applicable gene set enrichment for pathway analysis,” BMC Bioinformatics, vol. 10, no. 1, p. 161, May 2009, doi: 10.1186/1471-2105-10-161.

[7] W. Luo and C. Brouwer, “Pathview: an R/Bioconductor package for pathway-based data integration and visualization,” Bioinforma. Oxf. Engl., vol. 29, no. 14, pp. 1830–1831, Jul. 2013, doi: 10.1093/bioinformatics/btt285.

[8] O. Fornes et al., “JASPAR 2020: update of the open-access database of transcription factor binding profiles,” Nucleic Acids Res., vol. 48, no. D1, pp. D87–D92, Jan. 2020, doi: 10.1093/nar/gkz1001.
