# Supplementary material for: Transcriptional profiling reveals glucose-dependent regulation of COL13A1 mRNA in Pompe patients: Prospect for a novel disease mechanism
Source: Genes Dis. 2025 Jun 26;13(1):101738. doi: 10.1016/j.gendis.2025.101738 (PMC12495276; doi:10.1016/j.gendis.2025.101738)

## Supplementary Figure 1

Heat map of the differentially expressed genes, with a heat map of the expression levels of each gene in five tissues of interest as reported by GTEx Portal.


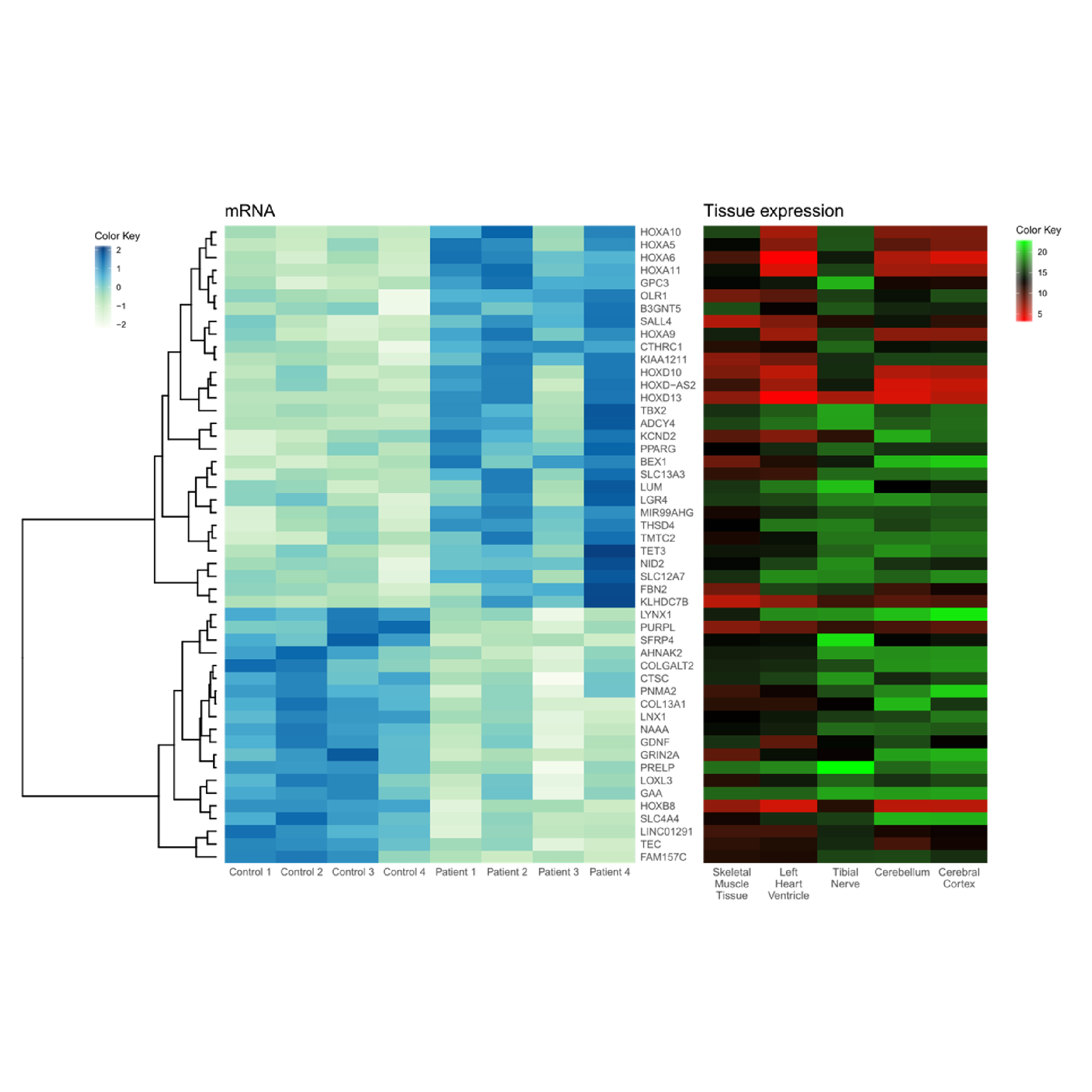

Supplement: Multimedia component 2 [file mmc2.docx]
