# Supplementary material for: Transcriptional profiling reveals glucose-dependent regulation of COL13A1 mRNA in Pompe patients: Prospect for a novel disease mechanism
Source: Genes Dis. 2025 Jun 26;13(1):101738. doi: 10.1016/j.gendis.2025.101738 (PMC12495276; doi:10.1016/j.gendis.2025.101738)

## Supplementary Figure 2

Validation of mRNA sequencing results in a larger cohort and a mouse muscle model.

RT-qPCR relative expression of selected genes in 10 controls and 16 patients, with a maintained differential expression of PIK3R1, COL13A1, GABRB3 and KCNMA1. (*: p<0.05, **: p<0.01, ***: p<0.001).


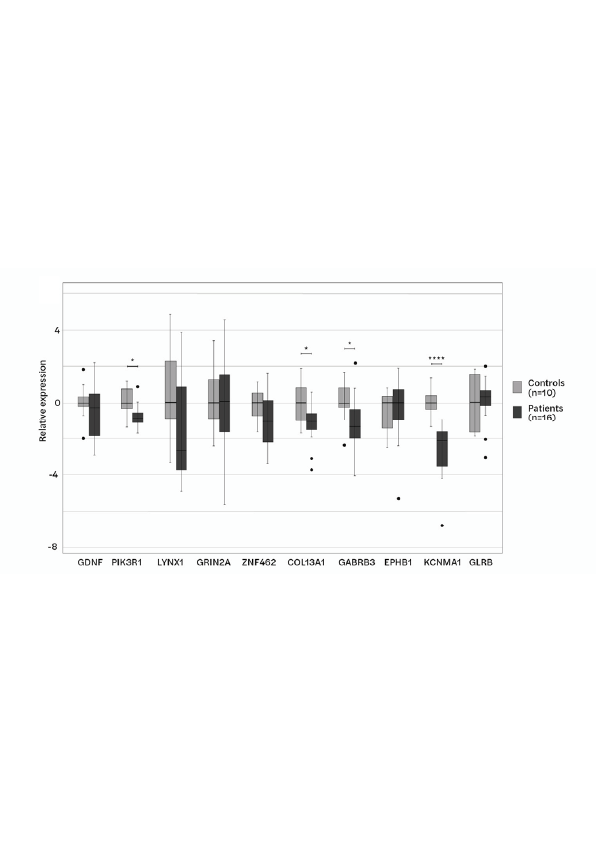

Supplement: Multimedia component 3 [file mmc3.docx]
