# Supplementary material for: Transcriptional profiling reveals glucose-dependent regulation of COL13A1 mRNA in Pompe patients: Prospect for a novel disease mechanism
Source: Genes Dis. 2025 Jun 26;13(1):101738. doi: 10.1016/j.gendis.2025.101738 (PMC12495276; doi:10.1016/j.gendis.2025.101738)

## Supplementary Figure 3

Transcription factor binding site enrichment analysis. Comparing the number of binding sites for each transcription factor in the promoters of differentially expressed genes in the Pompe mRNA sequencing dataset (blue lines) to 1000 random gene sets (black lines) of equal size. These show that unlike PPARgamma, none of the other transcription factors found in our dataset is enriched compared to 1000 random gene set, regardless of minimum match score.


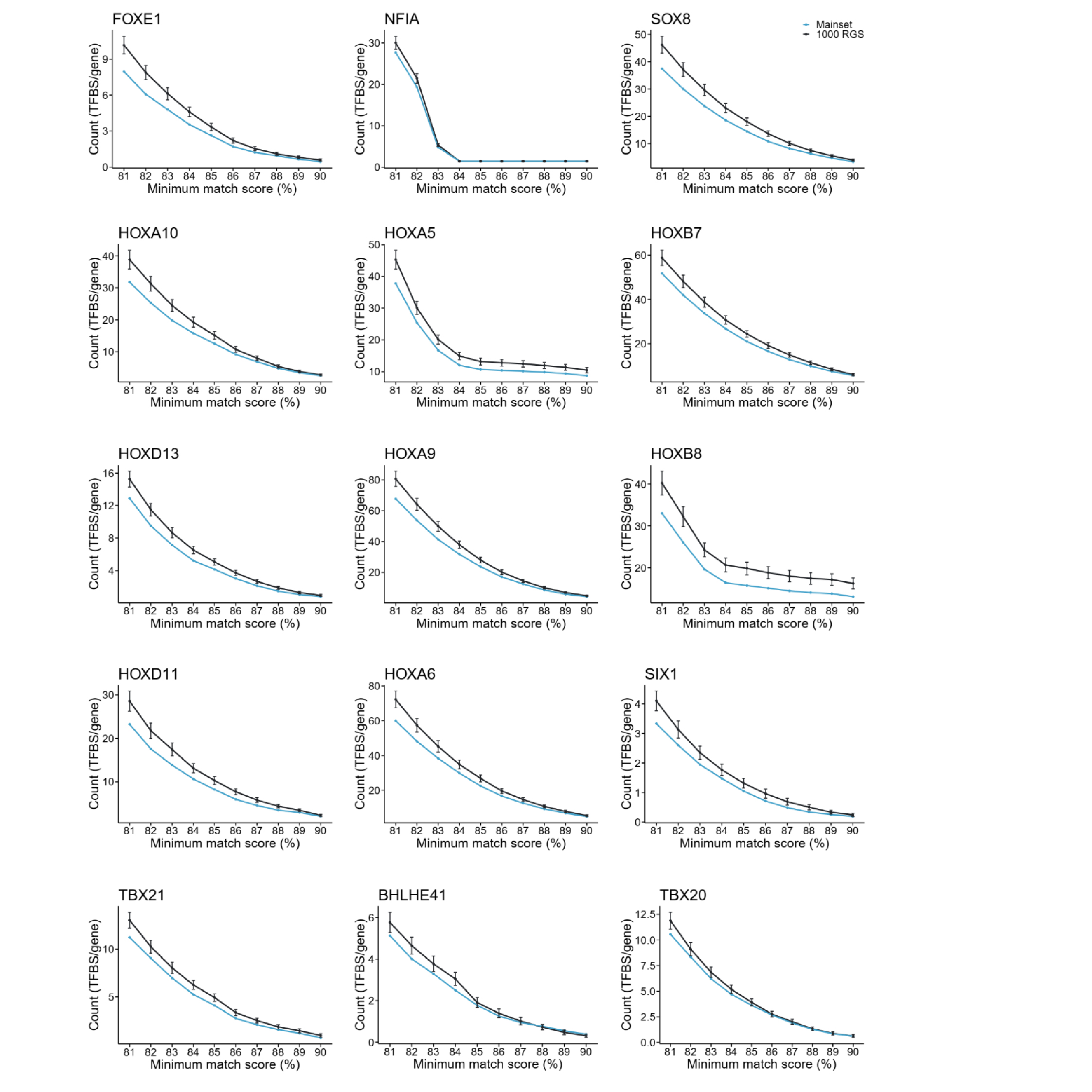

Supplement: Multimedia component 4 [file mmc4.docx]
