# Supplementary material for: Transcriptional profiling reveals glucose-dependent regulation of COL13A1 mRNA in Pompe patients: Prospect for a novel disease mechanism
Source: Genes Dis. 2025 Jun 26;13(1):101738. doi: 10.1016/j.gendis.2025.101738 (PMC12495276; doi:10.1016/j.gendis.2025.101738)
Supplement: Multimedia component 5 [file mmc5.docx]

## Supplementary: Table 1

**Selection of 10 differentially expressed genes based on their role in neuromuscular functioning**

| **Code** | **Name** | **Role** |
| --- | --- | --- |
| *GDNF* | Glial cell line-derived neurotrophic factor | Recombinant human GDNF can rescue avian and murine spinal motor neurons from programmed cell death. |
| *PIK3R1* | Phosphatidylinositol 3-kinase, regulatory subunit 1 | Pik3r1 -/- mice show increased glucose transport in skeletal muscle and adipocytes. |
| *LYNX1* | LY6/Neurotoxin 1 | Lynx1 plays a role in maintaining the structure and function of adult neuromuscular junctions. |
| *GRIN2A* | Glutamate receptor, ionotrophic, n-methyl-d-aspartate, subunit 2A | Mono-allelic pathogenic variants are linked to a broad range of epilepsy syndromes, some of them associated with hypotonia. |
| *ZNF462* | Zinc finger protein 462 | Mono-allelic pathogenic variants are linked to Weiss-Kruszka syndrome, a multiple congenital anomaly syndrome with variable hypotonia. |
| *COL13A1* | Collagen, type XIII, alpha-1 | Bi-allelic pathogenic variants are linked to congenital myasthenic syndrome type 19, with hypotonia and muscle weakness. |
| *GABRB3* | Gamma-aminobutyric acid receptor, beta-3 | Mono-allelic pathogenic variants are linked to different types of epileptic syndromes, some of them with associated hypotonia. |
| *EPHB1* | Ephrin receptor EphB1 | The group of ephrin receptors and their ligands play a role in muscle contraction. |
| *KCNMA1* | Potassium channel, calcium-activated, large conductance, subfamily M, alpha member 1 | Mono-allelic pathogenic variants are linked to paroxysmal nonkinesigenic dyskinesia 3, with our without generalized epilepsy. Some patients present with hypotonia. |
| *GLRB* | Glycine receptor, beta subunit | Bi-allelic pathogenic variants are linked to hyperekplexia 2. Patients present with generalized stiffness at birth, intermittent tonic stiffening and hyperreflexia. |
