# Supplementary material for: Transcriptional profiling reveals glucose-dependent regulation of COL13A1 mRNA in Pompe patients: Prospect for a novel disease mechanism
Source: Genes Dis. 2025 Jun 26;13(1):101738. doi: 10.1016/j.gendis.2025.101738 (PMC12495276; doi:10.1016/j.gendis.2025.101738)
Supplement: Multimedia component 6 [file mmc6.docx]

## Supplementary: Table 2

List of primers used for RT-qPCRs

|  | **Gene** | **Forward primer 5’-3’** | **Reverse primer 5’-3’** |
| --- | --- | --- | --- |
| Housekeeping genes | *HMBS* | AAGAGTGTGGTGGGAACCAG | ACTGAACTCCTGCTGCTCGT |
|  | *B2M* | TGGAGGCTATCCAGCGTACT | TCAATGTCGGATGGATGAAA |
|  | *GAPDH* | CGAGATCCCTCCAAAATCAA | GGCAGAGATGATGACCCTTT |
| Genes of interest | *GDNF* | GGGCACCTGGAGTTAATGTC | AGCCACGACATCCCATAACT |
|  | *PIK3R1* | TTTGACTCTCCCGGATCTTG | TTTGACTCTCCCGGATCTTG |
|  | *LYNX1* | GTGTGTGCCTACAACGGAGA | TCCTGGTGGGGGTGTAGTAG |
|  | *GRIN2A* | TGGGACATGCAGAATGTGAT | AGAACAGCCTCGTCTTTGGA |
|  | *ZNF462* | TCTCAAGGCACACATTCAGG | GCAATGGCAAATTCATCCTT |
|  | *COL13A1* | CAATCAACTGCTGGACGAGA | CTGGTGGGCAGTTACATCCT |
|  | *GABRB3* | GACCGTTCAAAGAGCGAAAG | CAATGCCGCCTGAGACCT |
|  | *EPHB1* | TGAGGAGCATCACCTTGTCA | TCCTTGCTGTGTTGGTCTGA |
|  | *KCNMA1* | ATCTCTCCAGTGCCTTCGTG | CTCTCTCGGTTGGCAGACTT |
|  | *GLRB* | TGAAGCTCCCCAGTGATTTT | TGTTTTCCTGGGTCACATCA |
|  | *PPARG* | CGAAGACATTCCATTCACAAGA | ATGCAGGCTCCACTTTGATT |
|  | *FABP4* | CAGTGTGAATGGGGATGTGA | GGACACCCCCATCTAAGGTT |
| Murine housekeeping genes | *Hmbs* | TCGCATACAGACCGACACTG | GCCAGGGTACAAGGCTTTCA |
|  | *B2m* | ACGCCTGCAGAGTTAAGCAT | TCTCGATCCCAGTAGACGGT |
| Murine   genes of interest | *Gaa* | AACACCTCAGCCCACTCATG | AGAAGACACCGTGAGCCAAG |
|  | *Col13a1* | AGGATGTAACTGCCCACCAG | CTTTGATACCCACACGTCCA |
