# Supplementary material for: Transcriptional profiling reveals glucose-dependent regulation of COL13A1 mRNA in Pompe patients: Prospect for a novel disease mechanism
Source: Genes Dis. 2025 Jun 26;13(1):101738. doi: 10.1016/j.gendis.2025.101738 (PMC12495276; doi:10.1016/j.gendis.2025.101738)
Supplement: Multimedia component 7 [file mmc7.docx]

## Supplementary Table 3

Patient and control demographics and biochemical GAA activity. Transcript NM_000152

The patients are defined by age groups set as adult (≥ 18 years old), juvenile (< 18 years old), and infantile (≤ 1 year old). All reported variants are established pathogenic variants, associated with Pompe disease. Patients with an asterix were included in the initial mRNA sequencing experiment.

| **Patient** | **Gender** | **Age (y)** | **Age group** | **GAA enzymatic activity (nmol/mg/h)** | ***GAA* variants** | **Protein change** |
| --- | --- | --- | --- | --- | --- | --- |
| 1 | M | 51 | Adult | 14.5 | c.-32-13T>G  c.2261dupC | p.?  p.Val755Serfs*41 |
| 2 | M | 52 | Adult | 2.4 | c.-32-13T>G  c.2608C>T | p.?  p.Arg870* |
| 3 | F | 43 | Adult | 6.94 | c.-32-13T>G  c.525delT | p.?  p.Glu176Argfs*45 |
| 4 | M | 37 | Adult | 5.1 | c.-32-13T>G  c.1115A>T | p.?  p.His372Leu |
| 5 * | F | 0-1 | Infantile | 0 | c.854C>G  c.2331+2T>A | p.Pro285Arg  p.? |
| 6 | F | 35 | Adult | 5.2 | c.-32-13T>G  c.525delT | p.?  p.Glu176Argfs*45 |
| 7 | M | 1 | Infantile | 7.4 | c.-32-13T>G  c.2608C>T | p.?  p;Arg870* |
| 8 | F | 32 | Adult | 3 | c.-32-13T>G  c.2608C>T | p.?  p.Arg870* |
| 9 * | M | 0-1 | Infantile | 0 | c.854C>G  c.2331+2T>A | p.Pro285Arg  p.? |
| 10 | M | 16 | Juvenile | 3.5 | c.-32-13T>G  c.2237G>A | p.?  p.Trp746* |
| 11 | F | 0-1 | Infantile | 0 | c.1327-61_1437+171del  c.2331+2T>A | p.?  p.? |
| 12 * | F | 60 | Adult | 2.9 | c.-32-13T>G  c.2331+2T>A | p.?  p.? |
| 13 * | F | 30 | Adult | 2 | c.-32-13T>G  c.1121G>A | p.?  p.Cys374Tyr |
| 14 | F | 43 | Adult | 7.2 | c.-32-13T>G  c.2608C>T | p.?  p.Arg870* |
| 15 | F | 12 | Juvenile | 4.54 | c.-32-13T>G  c.634delG | p.?  p.Glu212Serfs*9 |
| 16 | M | 42 | Adult | 10.3 | c.-32-13T>G  c.2331+2T>A | p.?  p.? |
| C1 | M | 34 | Adult | 80.8 |  |  |
| C2 | M | 38 | Adult | 21 |  |  |
| C3 | F | 57 | Adult | 103 |  |  |
| C4 | F | 56 | Adult | 64.8 |  |  |
